# Sucralose Promotes Colitis-Associated Colorectal Cancer Risk in a Murine Model Along With Changes in Microbiota

**DOI:** 10.3389/fonc.2020.00710

**Published:** 2020-06-03

**Authors:** Xueting Li, Yuanli Liu, Yan Wang, Xue Li, Xinran Liu, Mengru Guo, Yiwei Tan, Xiaofa Qin, Xiuhong Wang, Mingshan Jiang

**Affiliations:** ^1^Department of Biochemistry and Molecular Biology, Heilongjiang Provincial Science and Technology Innovation Team in Higher Education Institutes for Infection and Immunity, Harbin Medical University, Harbin, China; ^2^GI Biopharma Inc., Westfield, NJ, United States; ^3^Department of General Surgery, The Second Affiliated Hospital of Harbin Medical University, Harbin, China

**Keywords:** sucralose, colitis-associated colorectal cancer, gut microbiota, digestive proteases, gut permeability

## Abstract

Sucralose is a calorie-free high-intensity artificial sweetener that is widely used in thousands of foods and beverages all over the world. Although it was initially regarded as a safe, inert food additive, its adverse effect on gut microbiota and health has drawn more and more attention as evidence accumulates. Studies by us and others revealed that sucralose exacerbated gut damage and inflammation in animal models for inflammatory bowel disease (IBD), including those for both ulcerative colitis, and Crohn's disease. Our study demonstrated that sucralose greatly aggravated dextran sulfate sodium (DSS)-induced colitis along with causing changes in gut microbiota, the gut barrier and impaired inactivation of digestive proteases mediated by deconjugated bilirubin. It is well-documented that IBD greatly increases the risk of colorectal cancer (CRC), the globally third-most-common cancer, which, like IBD, has a high rate in the developed countries. Azoxymethane (AOM)/DSS has been the most commonly used animal model for CRC. In this study, we further explored the effect of sucralose on tumorigenesis and the possible mechanism involved using the AOM/DSS mouse model. First, 1.5 mg/ml sucralose was included in the drinking water for 6 weeks to reach a relatively stable phase of impact on gut microbiota. Then, 10 mg/kg AOM was administered through intraperitoneal injection. Seven days later, 2.5% DSS was put in the drinking water for 5 days, followed by 2 weeks without DSS. The 5 days of DSS was then repeated, and the mice were sacrificed 6 weeks after AOM injection. The results showed that sucralose caused significant increases in the number and size of AOM/DSS-induced colorectal tumors along with changes in other parameters such as body and spleen weight, pathological scores, mortality, fecal β-glucuronidase and digestive proteases, gut barrier molecules, gut microbiota, inflammatory cytokines and pathways (TNFα, IL-1β, IL-6, IL-10, and TLR4/Myd88/NF-κB signaling), and STAT3/VEGF tumor-associated signaling pathway molecules. These results suggest that sucralose may increase tumorigenesis along with dysbiosis of gut microbiota, impaired inactivation of digestive protease, damage to the gut barrier, and exacerbated inflammation.

## Introduction

Colorectal cancer (CRC) is the third most commonly diagnosed cancer worldwide, with an incidence ranking third in men, only after lung and prostate cancer, and second in women, only after breast cancer (1). CRC is high in developed countries, and there is a trend of increase in multiple developing countries (2). Although an increase in screening is leading to a decline in CRC incidence in the elderly population, multiple studies in countries such as the USA, Canada, UK, Germany, Norway, Denmark, Sweden, Slovenia, Australia, and New Zealand have shown increases in CRC in those younger than 50 (2–4). A study using data on 143.7 million people aged 20–49 years from 20 European countries revealed an increase of CRC incidence from 2004/2005 to 2016 at 7.9% per year among subjects aged 20–29 years, 4.9% per year in the 30–39-years age group, and 1.6% per year in the 40–49-years age group (4). However, the cause for this remains a mystery.

Studies have found that CRC may be affected by multiple factors such as race and ethnicity, heredity, smoking, and alcohol (5). The high level of CRC in developed countries in North America, Europe, Australia, New Zealand, Japan, and South Korea suggested a close association between CRC and the modern lifestyle (2, 5). Intriguingly, inflammatory bowel disease (IBD), a devastating inflammatory disease of the gut, is also very high in developed countries (6, 7), and IBD greatly increased the risk of CRC with a hazard ratio as high as 33.3 (5), suggesting a likely intimate link between CRC and IBD (8–10). Recently, dysbiosis of microbiota in the development of IBD and CRC has drawn more and more attention (11–15). Accumulating evidence suggests that dysbiosis of gut microbiota, gut inflammation, and CRC may have intimate interaction and connection. There are reports that IBD and CRC may involve decreased abundance and diversity of gut bacteria along with a changed ratio of *Firmicutes, Bacteroidetes*, and *Proteobacteria* (14–16). Stool from patients with CRC had changes in the abundances of *Fusobacterium nucleatum, Peptostreptococcus anaerobius, Peptostreptococcus stomatis, Parvimonas micra, Solobacterium moorei*, and *Gemella morbillorum* (16, 17). An important question is what causes this dysbiosis of gut microbiota in modern society.

Not surprisingly, diet, not only in terms of nutrients but also of dietary chemicals, may have a huge impact on gut microbiota (11, 14, 15). In fact, it has been long proposed that food additives such as saccharin and sucralose may have played an important causative role in IBD due to their inhibition of gut bacteria and the resultant impaired inactivation of digestive proteases by deconjugated bilirubin through the action of bacterial β-glucuronidase (18, 19). Papers published in recent years in Nature showed that food additives such as artificial sweeteners and emulsifiers increased the risk of diabetes, obesity, and colitis through their adverse impact on gut bacteria (20, 21). Studies by us and others revealed that sucralose, the widely used artificial sweetener, increased the risk of IBD in animal models for both ulcerative colitis and Crohn's disease (22, 23). Our study showed that sucralose exacerbated dextran sulfate sodium (DSS)-induced colitis along with dysbiosis of gut microbiota, decrease in bacterial β-glucuronidase, increase in fecal digestive protease, and aggravated damage of the gut barrier and gut tissue. As azoxymethane (AOM)/DSS is the most commonly used animal model for CRC (24), and microbiota dysbiosis and barrier dysfunction may be a common ground for IBD and CRC (25, 26), we investigated the effect of sucralose on AOM/DSS-induced tumorigenesis in mice.

## Methods and Materials

### Animals

C57BL/6 mice (4 weeks old, from the Laboratory Animal Center of the Second Affiliated Hospital of Harbin Medical University) were adapted to the environment for 2 weeks before the experiment and were raised under specific pathogen-free conditions in the Animal Experimental Center of Harbin Medical University (24–25°C, humidity 70–75%, 12 h light/dark) with a standard diet and drinking water. All animal experiments strictly followed the requirements of animal feeding regulations of Harbin Medical University and met the ethical requirements for animal experiments.

### Chemicals and Reagents

N-benzoyl-L-tyrosine ethyl ester (BTEE), Na-Benzoyl-Larginine 4-nitroanilide hydrochloride (BAPNA), sucralose (≥98.0% HPLC), and Azoxymethane (AOM) were purchased from Sigma-Aldrich (St. Louis, MO, USA). 4-Nitrophenyl b-D-glucopyranoside was purchased from BBI Life Sciences. DSS (MW: 36–50 kDa) was obtained from MP Biomedical (Solon, OH, USA). The antibodies used in this study were anti-TLR4 (19811-1-AP, Proteintech), anti-VEGF (19003-1-AP, Proteintech), anti-STAT3 (10253-2-AP, Proteintech), anti-Phospho-Stat3 (Tyr705) (#9145, Cell Signaling Technology), anti-MyD88 (#4283, Cell Signaling Technology), anti-TRAF6 (YT4720, Immunoway), anti-inhibitor of NF-κB alpha (IκBα) (#4814, Cell Signaling Technology), and anti-Occludin (13409-1-AP, Proteintech), as well as anti-GAPDH, anti-β-Actin, goat anti-rabbit IgG, and goat anti-mouse IgG from ZSGB-BIO Co. Ltd. (Beijing, China). All other reagents used were of analytical grade.

### Treatment of Mice With Sucralose and AOM/DSS

**Figure 1:**
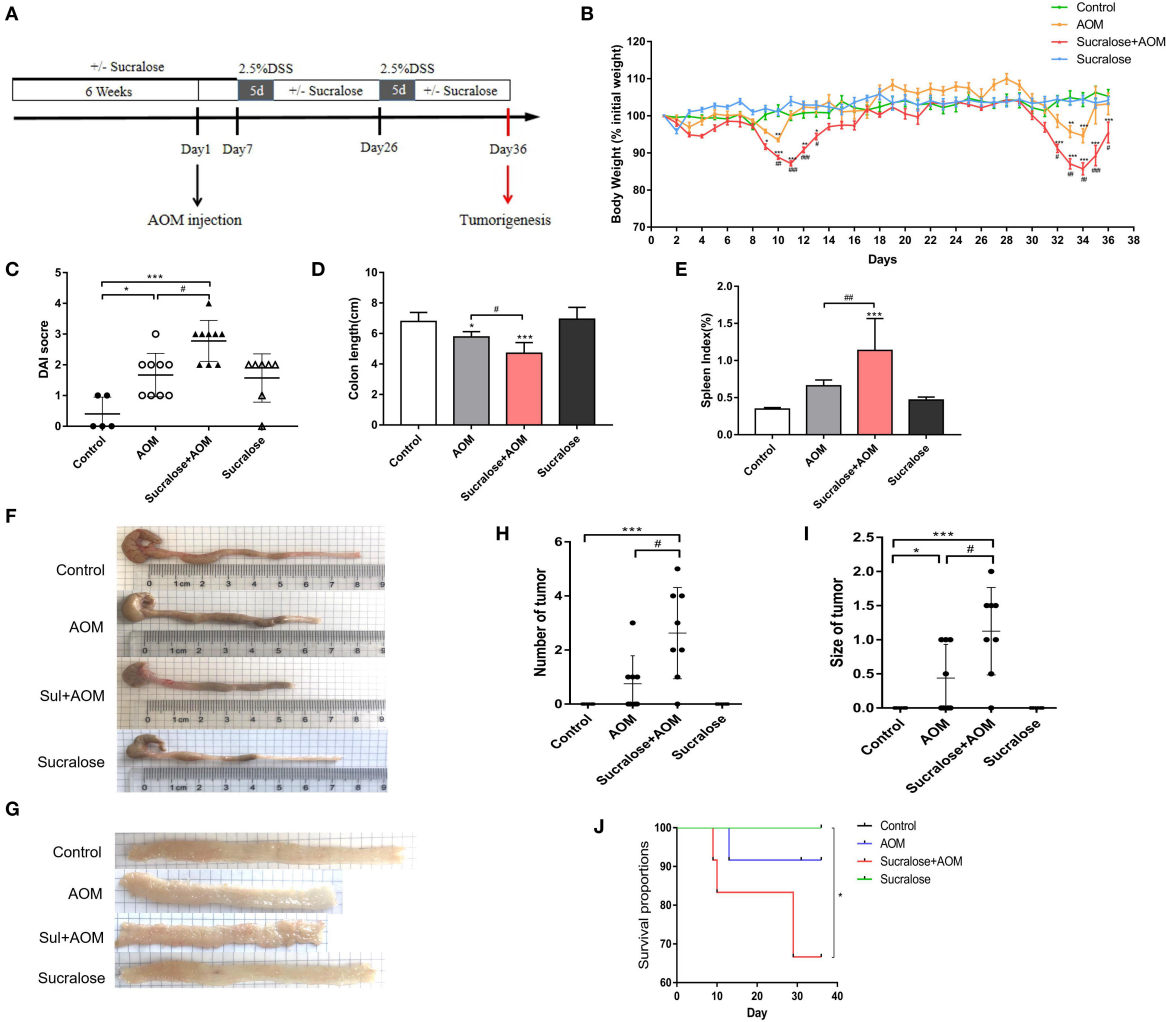
Sucralose significantly increases the severity of disease of AOM/DSS colon cancer mice. **(A)** Protocol of sucralose and AOM/DSS treatments used in this study. **(B)** Change in body weight, **(C)** disease activity index (DAI) score, **(D)** colon length, **(E)** spleen weight index, **(F)** photoraphs showing colon length, **(G)** photograph of colon tumor, (H) tumor number, **(I)** tumor size, and **(J)** survival rate. Compared with the control group: *P < 0.05, **P < 0.01, ***P < 0.001; compared with the AOM group: ^#^*P* < 0.05, ^*##*^*P* < 0.01, ^*###*^*P* < 0.001, *n* = 8.

Before the induction of CRC by AOM, sucralose was added to the drinking water of mice at 1.5 mg/ml for 6 weeks to induce explicit change in the gut microbiota (21, 27). Then, AOM was administered through intraperitoneal injection at 10 mg/kg. Seven days later, 2.5 % DSS was put in the drinking water for 5 days, followed by 2 weeks without DSS. The 5-days DSS was then repeated (28). Sucralose was given for the whole period, and the animals were sacrificed on the 36th day after AOM injection. An illustrated protocol can be seen in Figure 1A. Feces were collected just before the start of experiment and on day 1, 6, 25, and 36 after AOM injection. Gut, spleen, and blood were harvested at the end of the experiment. Samples were stored frozen prior to analysis.

### Disease Activity Index

The disease activity index (DAI) was calculated for each animal by adding together the scores for body weight, stool consistency, and stool blood as listed in Supplementary Table 1 and described in our previous studies (29).

### Tumor Analysis and Scoring of Colonic Damage

Tumor size was measured *ex vivo* according to the area against a standard background grid. Colonic tissues and tumors were fixed in 4% (w/v) paraformaldehyde and then paraffin-embedded, sectioned, and stained with hematoxylin and eosin (HE). Pathological changes were observed and scored. Colonic damage was graded in a blinded manner, as described in Supplementary Table 2 and in our previous studies (27).

### Determination of Fecal β-Glucuronidase, Trypsin, and Chymotrypsin Activity

Feces were collected, added to PBS containing PMSF, and homogenized. After centrifuging, the supernatant was collected for the assay. β-glucuronidase, trypsin, and chymotrypsin (amidase) activities were measured by spectrophotometry using 4-Nitrophenyl b-D-glucopyranoside, Na-Benzoyl-L-arginine 4-nitroanilide hydrochloride (BAPNA), and N-benzoyl-l-tyrosine ethyl ester (BTEE) as the substrate, respectively, according to the methods described in detail in our previous studies (27, 30).

### Colon RNA Extraction and qRT-PCR

Total RNA was isolated from colon tissues using the UNlQ-10 Column Trizol Total RNA Isolation Kit (Sangon Biotech, Shanghai, China). The RNA concentration and OD260/280 absorbance ratio were then measured using a Nanodrop ND-1000 Spectrophotometer (Thermo Fisher Scientific, Waltham, MA, USA). The RNA was transcribed into cDNA using 5X All-In-One RT MasterMix (abm, Jiangsu, China; cat. no. G492). Quantitative real-time polymerase chain reaction (qRT-PCR) was performed in volumes of 20 μl containing 1 μl of each primer (Supplementary Table 3) in the FastStart Universal SYBR Green Master (Roche, Basel, Switzerland; cat.no.04913850001) according to the manufacturer's instructions. PCR amplification was performed with the following conditions: 30 s at 95°C, followed by 40 cycles of 5 s at 95°C and 31 s at 60°C. After that, a melting curve analysis was performed to confirm the specificity of the qRT-PCR. All samples were analyzed in triplicate, and the results were normalized to the expression of GAPDH. The results were calculated by the 2^−ΔΔCt^ equation.

### Analysis of Fecal Bacteria

Feces on day 36 were used to detect changes in the intestinal flora of the mice. Bacterial DNA in 0.15 g of feces collected by the end of the experiment was extracted by TIANamp Stool DNA Kit (TIANGEN, China) then amplified by qPCR (Applied Biosystems 7500 PCR) using the primers listed in Supplementary Table 4. The abundance of total bacteria in feces was assessed as the relative ratio of 16S RNA (16S) to DNA, while the abundance of certain bacteria was calculated as the relative ratio of the specific sequence to 16S.

### ELISA and Western Blot

Segments of colon were homogenized using RIPA buffer and protein inhibitor cocktail (1:10) (PhosSTOP ESAYpack, Roche). The homogenates were kept on ice for 30 min and centrifuged at 12,000 g for 5 min at 4°C. The protein concentration was determined using the BCA Protein Assay Kit (Beyotime, Shanghai, China). For ELISA assays, colon tissue protein was collected and assayed for IL-1β, IL-10, IL-6, and MPO, blood was collected, and D-Lac in serum was measured according to the manufacturer's instructions. The results for IL-1β, IL-10, and IL-6 are expressed in pg/μg, MPO is expressed in U/mg, and D-Lac is expressed in nmol/L. For Western blot analysis, 20–80 μg of protein was electroblotted onto a PVDF membrane after separation by 10% SDS-polyacrylamide gel electrophoresis. Immunoblots were incubated with primary antibodies against Occludin, STAT3, p-STAT3, VEGF, TNFα, TLR4, MyD88, TRAF6, NF-κB inhibitor alpha (IκBα), and GAPDH or β-Actin. Chemiluminescent signals were analyzed using the Quantity One (version 4.5.2) program (Bio-Rad Laboratories) and Image J software.

### Statistical Analysis

The experimental data were plotted and analyzed with Graphpad Prism version 7.0, and the experimental results were expressed as mean ± standard error (*n* = 5–10 in each group). The results of the experiments were analyzed using repeated-measures ANOVA with Greenhouse-Geisser correction and Tukey's multiple comparisons test or *t*-test. *P* < 0.05 being regarded as significant difference. Pearson correlation coefficient was used for correlation analysis, with *P* < 0.05 being considered statistically significant.

## Results

### Effect of Sucralose on Some General Parameters of the Body and Colon and Colorectal Tumors in the AOM/DSS Model

Figure 1A illustrates the protocol of treatment in this study. We can see some fluctuations in body weight in the AOM/DSS groups (Figure 1B), which would be mainly due to the repeated treatment and withdraw of DSS. DSS will result in colitis along with diarrhea, bloody stool, rapid weight loss, inflammation and shorting of the colon, which may gradually recover after withdrawal. Compared with mice treated with AOM/DSS alone, the AOM/DSS + sucralose group showed more severe weight loss, more blood in stool, and a significantly lower DAI score, more shortening of the colon, and higher spleen weight (Figures 1B–F). Sucralose also increased the number and size of tumors induced by AOM/DSS, with a positive rate of 87.5% in the AOM/DSS + sucralose group vs. 50% in the AOM/DSS-alone group (Figures 1G–I). In addition, the AOM/DSS + sucralose group also showed higher mortality (Figure 1J).

### Effects of Sucralose on the Activity of Digestive Protease, β-Glucuronidase, and the Gut Barrier

We measured the digestive proteases trypsin and chymotrypsin and β-glucuronidase in feces, MUC2 and tight junction molecules (Occludin, ZO-1, claudin-1, and claudin-4) in the mucosa, and D-Lac in serum (a bacterial product of the gut that infiltrates into the blood) as a parameter for gut permeability.

**Figure 2:**
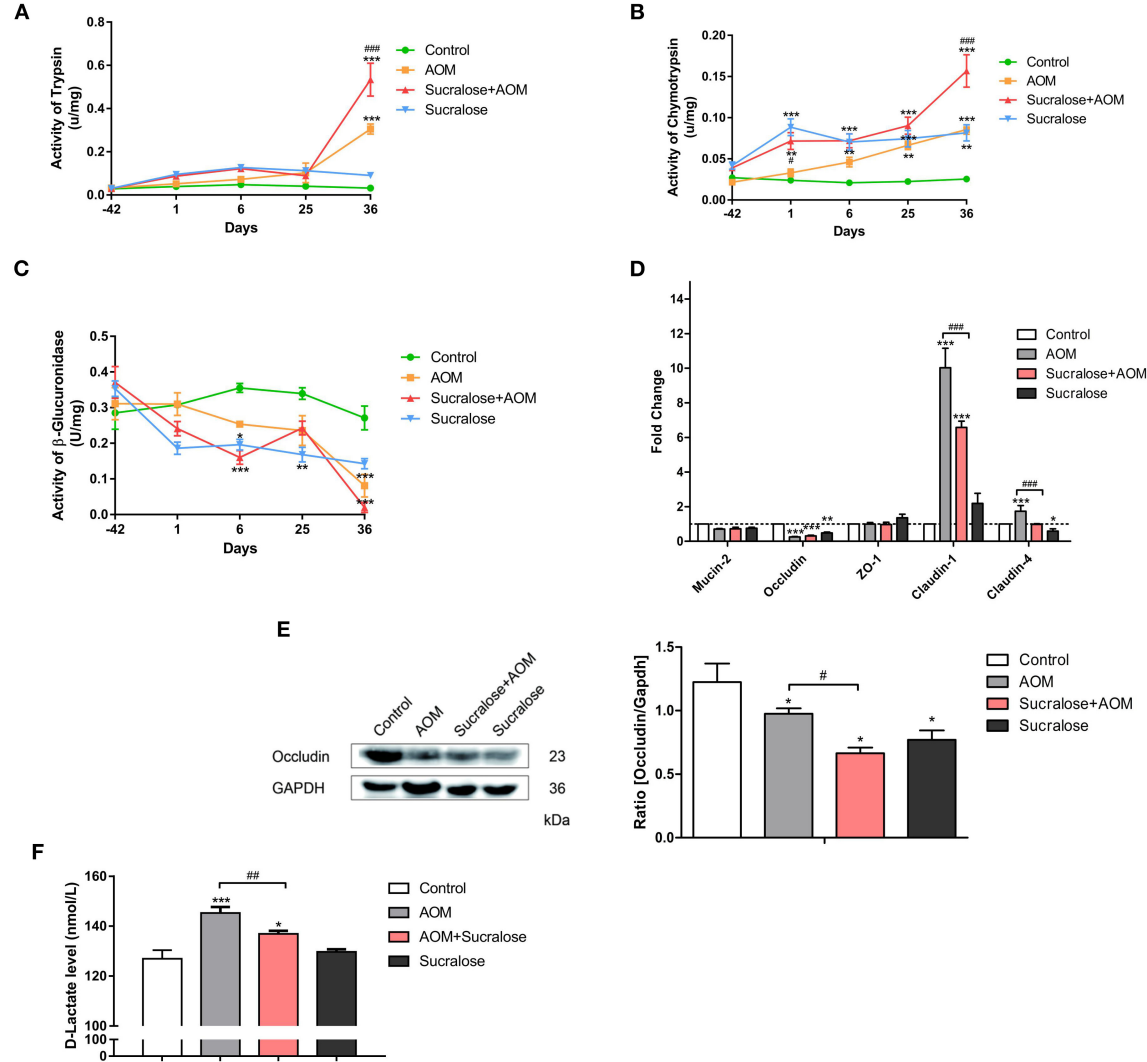
Sucralose aggravates damage to the colonic mucosal barrier in AOM/DSS colon cancer mice. **(A)** Trypsin activity in feces, **(B)** chymotrypsin activity in feces, **(C)** β-glucuronidase activity in feces, **(D)** changes in mucosal barrier molecule mRNA levels, **(E)** occluding (tight junction protein) expression, and **(F)** serum D-Lac levels. Compared with the control group: *P < 0.05, **P < 0.01, ***P < 0.001; compared with the AOM group: ^#^*P* < 0.05, ^*##*^*P* < 0.01, ^*###*^*P* < 0.001, *n* = 8.

The results showed that mice treated with AOM/DSS alone demonstrated significantly higher levels of fecal trypsin and chymotrypsin but a lower level of fecal β-glucuronidase, as well as exhibiting reduced occludin and increased claudin-1 and claudin-4 in muscosa and elevated serum D-Lac (Figure 2), suggesting damage to the gut barrier and increased gut permeability. AOM/DSS + sucralose caused further increase in fecal trypsin and chymotrypsin and decrease in fecal β-glucuronidase, as well as increased mucosal occludin, claudin-1, and claudin-4, suggesting further damage to the gut barrier (Figure 2). β-glucuronidase showed a significant negative correlation with trypsin and chymotrypsin (Table 1). Surprisingly, the serum D-Lac level of the AOM/DSS + sucralose group, although significantly higher than control, is significantly lower than that of the AOM/DSS group, which will be discussed in detail in the next section.

**Table 1:** Correlation between β-glucuronidase, trypsin and chymotrypsin.

	**β-glucuronidase**	
	***r***	***p-*value**
Trypsin	**−0.7066**	0.0001
Chymotrypsin	**−0.6683**	0.0001

*Bold values indicate that the two are significantly correlated*.

### Effects of Sucralose on Colonic Pathological Change

**Figure 3:**
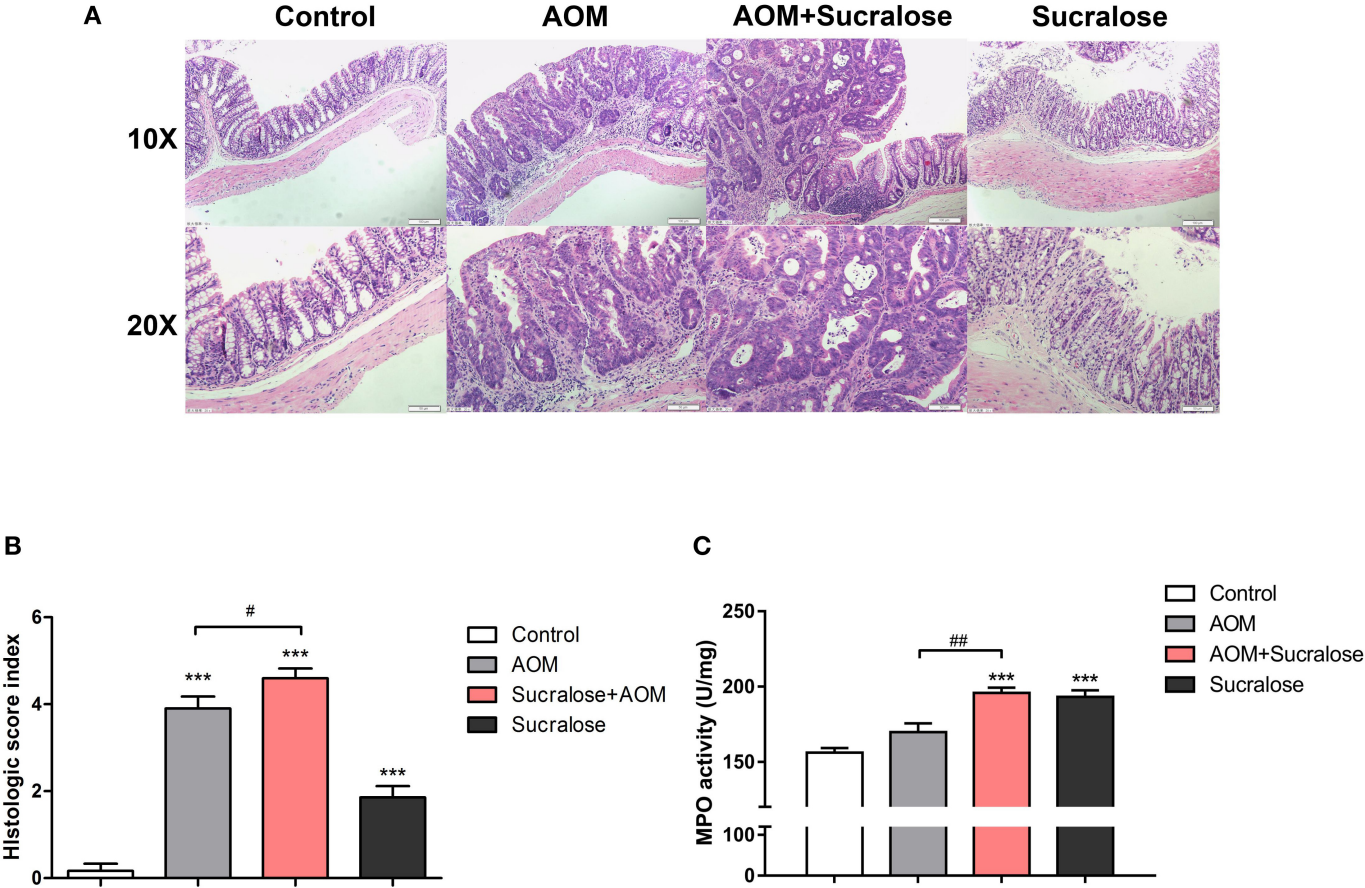
Sucralose significantly increases colonic tissue damage in AOM/DSS colon cancer mice. **(A)** Light microscopy evaluation of hematoxylin and eosin (H&E)-stained sections and **(B)** histological score (*n* = 5, scale bar = 50 μm). **(C)** MPO activity of colon tissue (*n* = 5). Compared with the control group: ***P < 0.001; compared with the AOM group: ^#^*P* < 0.05, ^*##*^*P* < 0.01, *n* = 8.

AOM/DSS caused marked epithelial destruction, infiltration of inflammatory cells, crypt deformation, increased abundance of mitotic cells, and a high degree of dysplasia, indicating the existence of inflammation and highly differentiated adenocarcinoma of the colon (Figure 3A), along with increases in microscopic lesion scores (Figure 3B) and MPO activity (Figure 3C). Treatment with sucralose caused even more pronounced changes than did AOM/DSS alone.

### Effect of Sucralose on the Composition of Fecal Microbiota

**Figure 4:**
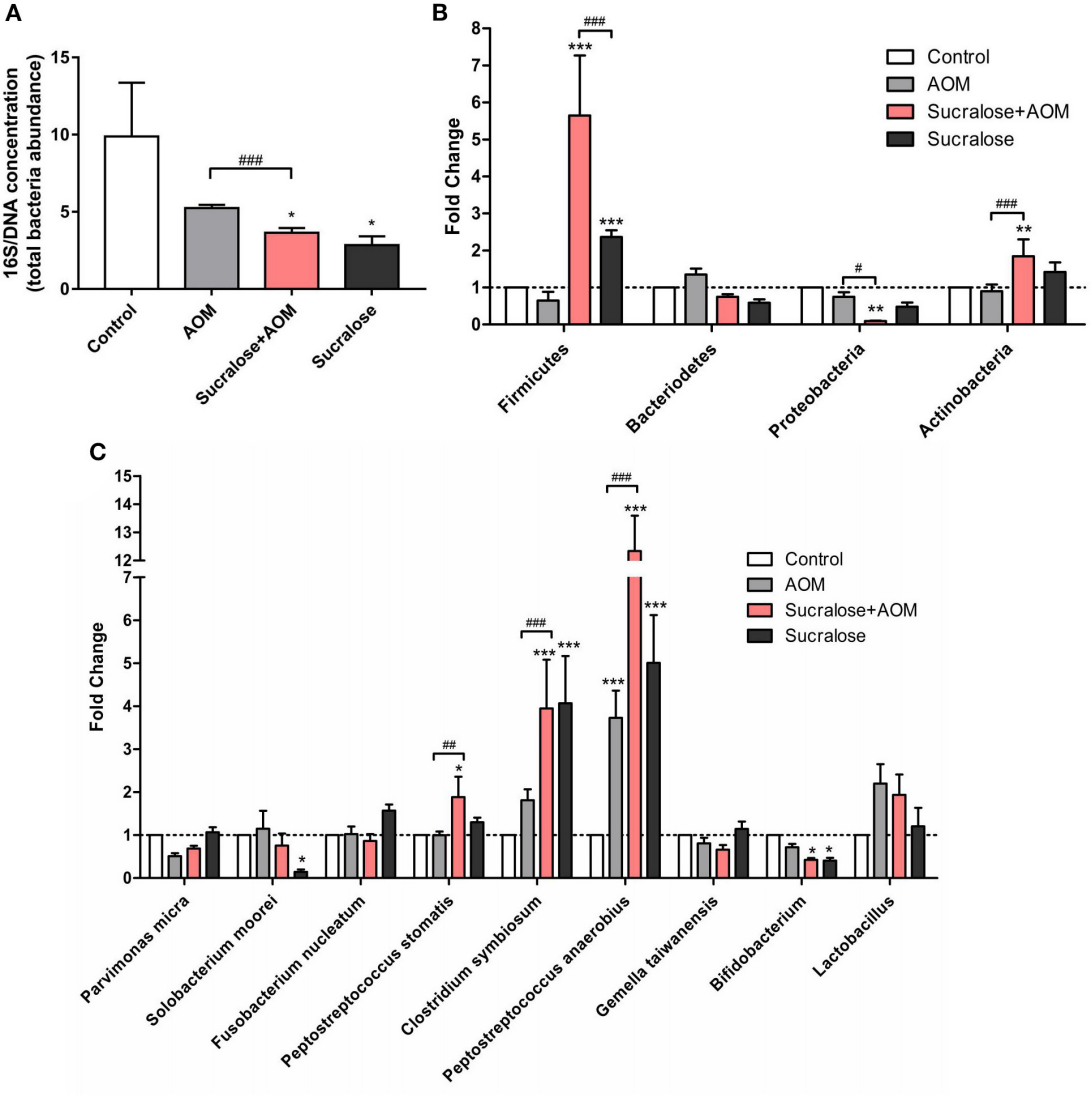
Sucralose alters mouse fecal microbiota composition. **(A)** Changes in 16S/DNA concentration. **(B)** Changes in Firmicutes, bacteriodetes, proteobacteria, and actinobacteria. **(C)** Changes in *Parvimonas micra, Solobacterium moorei, Fusobacterium nucleatum, Peptostreptococcus stomatis, Clostridium symbiosum, Gemella*, and *Peptostreptococcus anaerobius*. Compared with the control group: *P < 0.05, **P < 0.01, ***P < 0.001; compared with the AOM group: ^#^*P* < 0.05, ^*##*^*P* < 0.01, ^*###*^*P* < 0.001, *n* = 8.

We assessed the abundances of total gut bacteria and of the dominant phyla, as well as of some of the species that have been reported to be associated with CRC. Compared to control, all the three AOM/DSS and sucralose treated groups showed significant decreases in bacterial abundance as measured by 16S/DNA (Figure 4A). Treatment with AOM/DSS alone caused a significant increase in Peptostreptococcus anaerobius, while the sucralose-only group showed significant increases in Firmicures, Clostridium symbiosum, and Peptostreptococcus anaerobius and decreases in Solobacterium moorei and Bifidobacteria as compared to control. Compared to the AOM/DSS only group, AOM/DSS + sucralose caused significant increases in *Ficmicures, Actinomycetes, Peptostreptococcus stomatis, Clostridium symbiosum*, and *Peptostreptococcus anaerobius* and a decrease in proteobacteria (Figures 4B,C). Table 2 shows the correlations among the changes in gut microbiota and β-glucuronidase, trypsin, and chymotrypsin. β-glucuronidase showed significant positive correlations with 16S/DNA, Proteobacteria, Gemella taiwanensis, Parvimonas micra, and Bifidobacterium and significant negative correlations with *Firmicutes, Lactobacillus*, and *Peptostreptococcus anaerobius*. Trypsin showed significant positive correlations with *Firmicutes, Actinobacteria, Clostridium symbiosum, Peptostreptococcus anaerobius, Peptostreptococcus stomatis*, and *Solobacterium moorel* and significant negative correlations with *Proteobacteria*. *Gemella taiwanensis*, and *Parvimonas micra*. Chymotrypsin showed significant positive correlations with *Firmicutes, Actinobacteria, Clostridium symbiosum, Lactobacillus, Peptostreptococcus anaerobius*, and *Peptostreptococcus stomatis* and significant negative correlations with 16s/DNA and *Proteobacteri*.

**Table 2:** Correlation between CRC-associated bacterial abundances and trypsin, chymotrypsin, and β-glucuronidase.

**Bacteria**	**Trypsin**		**Chymotrypsin**		**β-glucuronidase**	
	***r***	***p*-value**	***r***	***p*-value**	***r***	***p*-value**
Peptostreptococcus anaerobius	**0.4968**	0.0038	**0.5437**	0.0013	**−0.4599**	0.0081
Clostridium symbiosum	**0.4794**	0.0055	**0.6692**	0.0001	−0.273	0.1305
Peptostreptococcus stomatis	**0.7415**	0.0001	**0.8609**	0.0001	−0.1962	0.2817
Bifidobacterium	−0.2603	0.1503	−0.3168	0.0773	**0.6513**	0.0001
Solobacterium moorei	**0.4531**	0.0092	0.2859	0.1127	0.1316	0.4728
Lactobacillus	0.2774	0.1451	**0.7676**	0.0001	**−0.3733**	0.0461
Gemella taiwanensis	**−0.3718**	0.0362	−0.2367	0.1922	**0.4309**	0.0138
Parvimonas micra	**−0.3626**	0.0414	−0.1284	0.4837	**0.5559**	0.0010
Fusobacterium nucleatum	−0.1716	0.3477	−0.02862	0.8764	0.1917	0.2934
Actinobacteria	**0.5001**	0.0247	**0.6457**	0.0021	−0.3064	0.1888
Bacteriodetes	0.1051	0.6591	−0.1642	0.4891	0.1103	0.6434
Proteobacteria	**−0.5887**	0.0063	**−0.7221**	0.0003	**0.7044**	0.0005
Firmicutes	**0.6514**	0.0019	**0.7703**	0.0001	**−0.4866**	0.0296

*Bold values indicate that the two are significantly correlated*.

### Effect of Sucralose on the TLR4/MyD88/TRAF6/NF-B Pathway and Inflammatory Cytokines of the Colon

We measured a variety of inflammatory cytokines as well as key molecules of the TLR4/MyD88/TRAF6/NF-κB pathway associated with gut inflammation by increased infiltration of components from gut bacteria.

**Figure 5:**
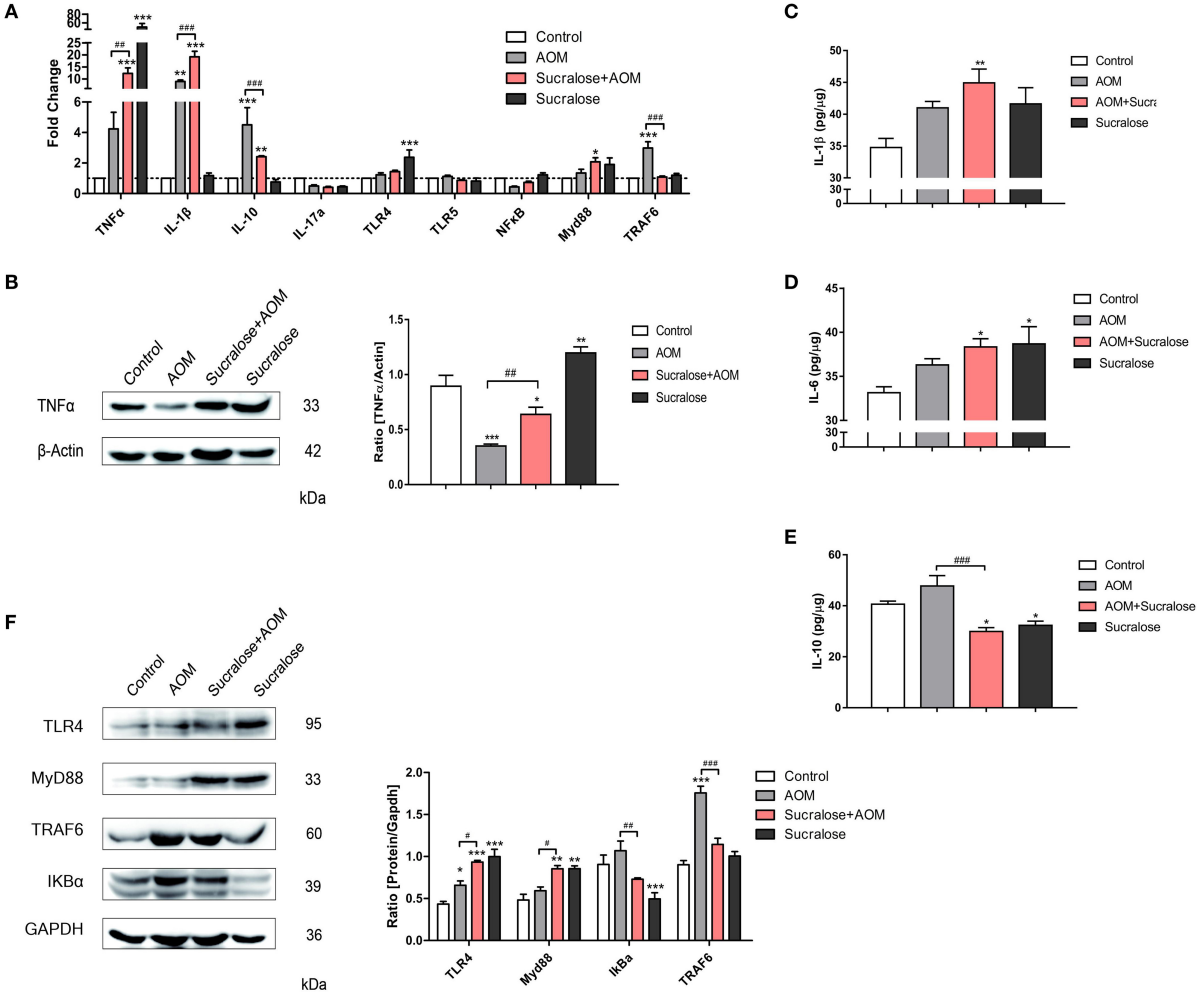
Sucralose promotes expression of pro-inflammatory factors. **(A)** Changes in mRNA levels of cytokines and TLR4/MyD88/TRAF6/NF-κB signaling pathways (*n* = 8). **(B)** Protein expression of TNFα. **(C)** Proteins of IL-1β in colon tissues. **(D)** Protein levels of IL-6 in colon tissue. **(E)** Protein levels of IL-10 in colon tissues. **(F)** Changes in protein levels of TLR4/MyD88/TRAF6/NF-κB signaling pathways. Compared with the control group: *P < 0.05, **P < 0.01, ***P < 0.001; compared with the AOM group: ^#^*P* < 0.05, ^*##*^*P* < 0.01, ^*###*^*P* < 0.001, *n* = 5.

In terms of mRNA expression (Figure 5A), the AOM/DSS group showed significant increases in IL-1β, IL-10, and TRAF6 vs. control. Sucralose alone also resulted in significantly increased expressions of TNFα and TLR4. Compared to the AOM/DSS-alone group, the AOM/DSS + sucralose group showed significantly higher expressions of TNFα and IL-1β and lower levels of IL-10 and TRAF6.

As for the protein level by Western blot assay (Figures 5B–F), the AOM/DSS group showed significant increases in IL-6, TLR4, and TRAF6 but a lower level of TNFα vs. control. Sucralose alone also resulted in significantly increased expressions of TNFα, TLR4, and Myd88 but decreases in IL-10 and IκBα. Compared to the AOM/DSS-alone group, the AOM/DSS + sucralose group showed significantly higher levels of TNFα, TLR4, and Myd88 but lower levels of IL-10, IκBα, and TRAF6.

### Effect of Sucralose on STAT3-VEGF Signaling of Colon Tissue

**Figure 6:**
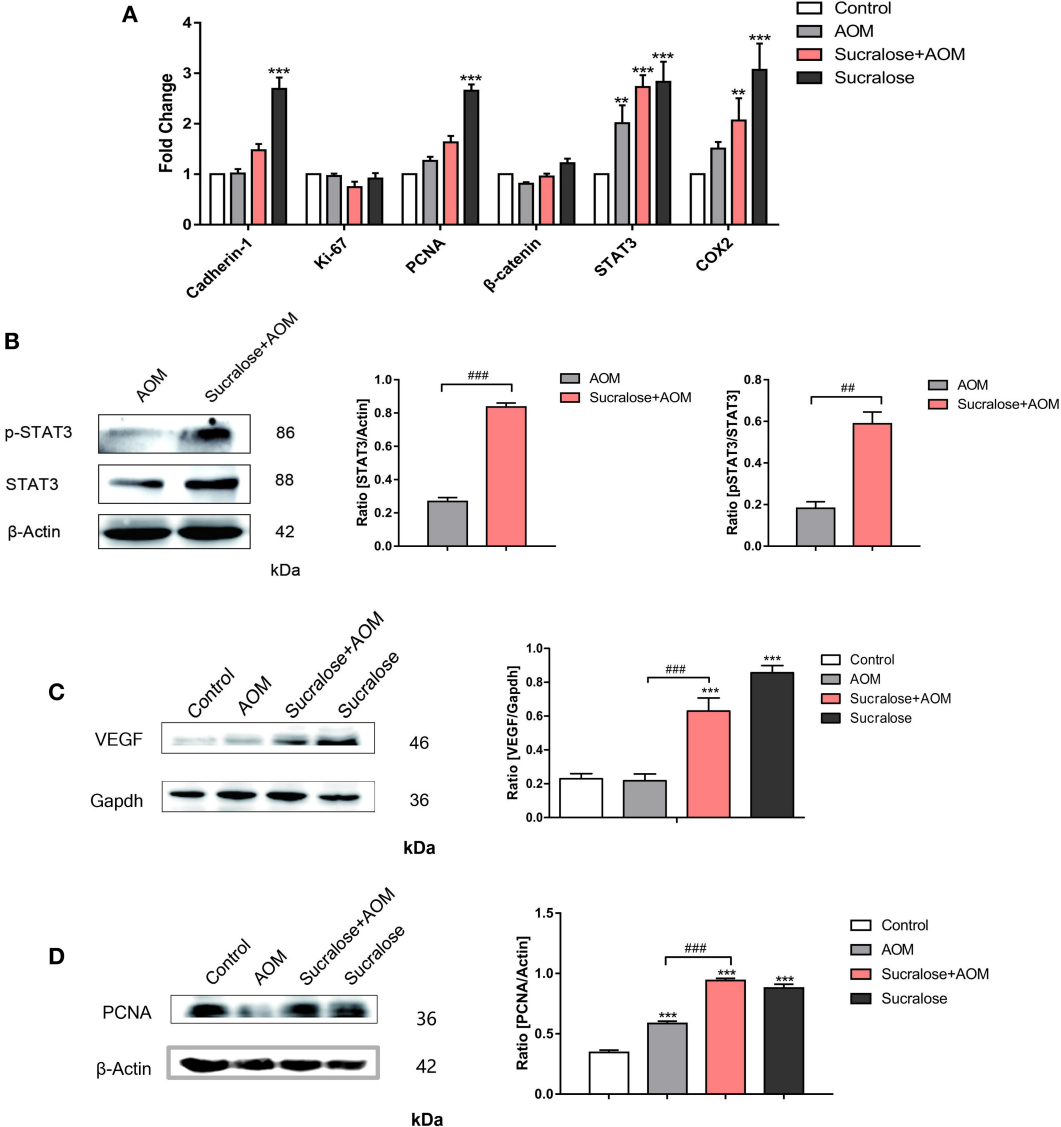
Sucralose promotes tumor-associated gene expression. **(A)** mRNA levels of tumor-associated genes. **(B)** Changes in STAT3 and pSTAT3 in tumor tissues. **(C)** Changes in VEGF protein in colon tissue. **(D)** Expression of PCNA protein in colon tissues. Compared with the control group: **P < 0.01, ***P < 0.001; compared with the AOM group: ^*##*^*P* < 0.01, ^*###*^*P* < 0.001, *n* = 5.

We analyzed the mRNA level of tumor-associated genes such as Cadherin-1, Ki-67, PCNA, β-catenin, STAT3, and COX2 (Figure 6A), as well as the protein levels of tumor marker VEGF, its downstream transcription activator STAT3 and p-STAT3, and the tumor proliferation marker PCNA (Figures 6B–D). AOM/DSS did not show an effect on VEGF but increased the level of PCNA. Compared to AOM/DSS alone, the AOM/DSS + sucralose group showed significant increases in VEGF, STAT3, p-STAT3, and PCNA. Sucralose alone showed a high level of VEGF and PCNA.

## Discussion

From the results above, we can see that sucralose caused significant increases in the number and size of AOM/DSS-induced colorectal tumors. A likely mechanistic explanation would be that inflammation was exacerbated by sucralose, as demonstrated by the higher expression or protein levels of TNFα and IL-1β, decrease in IL-10, and increased weight of spleen in the AOM/DSS + sucralose group compared to the AOM/DSS-alone group. Inflammation serves as a critical link between IBD and CRC (10). Interestingly, despite more damage to the gut barrier, as demonstrated by the reduced mucosal occludin, claudin-1, and claudin-4, the AOM/DSS + sucralose group had a lower level of serum D-lactate, a product of gut bacteria, than the AOM/DSS-alone group. This discrepancy may likely be due to the significant reduction in gut bacteria by sucralose shown in this study (Figure 4A) as well as in a study by others that sucralose can cause general inhibition of both total anaerobes and aerobic bacteria (25). This suggests the exacerbated inflammation in the sucralose group may be caused by factors other than increased infiltration of bacterial components. As AOM/DSS + sucralose caused greater increases in fecal trypsin and chymotrypsin, we propose that more severe damage to the gut barrier and increased infiltration of other toxicants such as DSS are the result of more severe impaired inactivation of digestive proteases and that changes in gut microbiota may have played a critical role.

Under normal conditions, digestive proteases such as trypsin and chymotrypsin are present at very low levels in feces (31–34). This inactivation of digestive proteases depends on gut bacteria, as a large amount of digestive proteases appear in the lower gut when animals are raised under germ-free conditions (35) or are treated with antibiotics (36) or saccharin and sucralose (37, 38). Both our *in vitro* and *in vivo* studies showed inhibition of digestive proteases by unconjugated but not by conjugated bilirubin (39, 40). Thus, deconjugation of bilirubin by the bacterial β-glucuronidase enriched in certain kinds of gut bacteria may be the key link between gut bacteria and the inactivation of digestive proteases in gut lumen. This also provided an explanation of how a reduction in gut bacteria can result in more damage and pathogenesis of the gut, which is also shown in other multiple studies. For instance, a study by Zhan et al. found that germ-free mice developed significantly more and larger tumors compared with conventional specific pathogen-free (SPF) mice after AOM and DSS treatment despite the lack of early acute inflammation (41). The germ-free mice showed a delay in intestinal epithelial repair of DSS-induced injury, resulting in a late onset of proinflammatory and protumorigenic responses and increased epithelial proliferation and microadenoma formation. In another study, it was shown that germ-free mice had enhanced hemorrhaging, epithelial injury, and mortality as a consequence of a weakened intestinal barrier despite only minimal inflammation (42). Impairment of the deconjugated bilirubin-mediated inactivation of digestive proteases due to a reduction in or lack of gut bacteria, and thus of the bacterial β-glucuronidase needed for bilirubin deconjugation, may be a shared key mechanism for all these mysteries (43). This notion is strongly supported by the significant negative correlation between β-glucuronidase and trypsin and chymotrypsin shown in Table 1 as well as the significant positive correlation between gut bacteria abundance and fecal β-glucuronidase and significant negative correlation between gut bacteria abundance and digestive proteases such as chymotrypsin shown in Table 2. Despite this, as shown in Table 2, although gut bacteria such as Firmicutes at the phylum level showed significant negative correlations with β-glucuronidase and negative correlations with trypsin and chymotrypsin, the lower level of gut bacteria of the Firmicutes phylum such as Gemella taiwanensis, *Lactobacillus, Peptostreptococcus anaerobius*, and *Parvimonas micra* may have an opposite correlation. Studies have shown that even the same species of gut bacteria may have different impacts. For instance, the enterotoxigenic strain of *B. fragilis* may serve as a trigger for colitis and tumorigenesis, while the non-toxigenic strain of *B. fragilis* may serve as an anti-inflammatory probiotic and protect against colitis and CRC (11). Thus, it remains a formidable task to elucidate the association between dysbiosis of gut bacteria and diseases.

To date, approval of food additives has never taken into consideration their impact on gut microbiota and related health. However, overwhelming evidence has shown the critical role of gut microbiota dysbiosis in the dramatic increases of many diseases in modern society (44, 45). In view of the synergistic effect of bacterial glycosidase and pancreatic digestive proteases on the degradation of the protective mucus layer, the frequent, constant use of food additives such as sucralose and saccharin may be more detrimental to gut microbiota and health than antibiotics (46). We may need to adopt a prudent attitude to claiming the absolute safety of currently approved food additives for gut microbiota and health.

IBD and CRC and many other diseases in modern society are multifactorial. Nevertheless, given the substantial synergistic effect of AOM and DSS in the model used in this study as well as the added effect of sucralose on AOM/DSS, there is a major problem with basing safety evaluation on separate assessments of individual compounds. The many kinds of food additives in processed food will have added effects on gut microbiota (47). This remains a major challenge, and there is a long way to go to achieve a precise evaluation of the many environmental factors associated with modernization and to find out the causes and mechanisms of diseases emerging and dramatically increasing in modern society. We hope that this preliminary early-stage study may spur more research in this area.

## Data Availability Statement

All datasets generated for this study are included in the article/Supplementary Material.

## Ethics Statement

The animal study was reviewed and approved by Institutional Research Board of Harbin Medical University.

## Author Contributions

XuetL, YL, YW, XueL, XiL, MG, and YT conducted the experiments. XuetL, XW, and XQ wrote the manuscript. WX and MJ contributed to study supervision. All authors participated in the design, interpretation of the studies and analysis of the data, and review of the manuscript.

## Conflict of Interest

XQ was employed by the company GI Biopharma Inc. The remaining authors declare that the research was conducted in the absence of any commercial or financial relationships that could be construed as a potential conflict of interest.
