# Supplementary material for: Sucralose Promotes Colitis-Associated Colorectal Cancer Risk in a Murine Model Along With Changes in Microbiota
Source: Front Oncol. 2020 Jun 3;10:710. doi: 10.3389/fonc.2020.00710 (PMC7286428; doi:10.3389/fonc.2020.00710)
Supplement: Supplementary file 1 [file Data_Sheet_1.docx]

**Supplementary Table 1.** The criteria of scoring DAI

| Score | Weight loss (%) | Stool consistency | Occult/gross bleeding |
| --- | --- | --- | --- |
| 0 | None | Normal | Normal |
| 1 | 1-5 |  |  |
| 2 | 5-10 | Loose stools | Hemoccult positive |
| 3 | 10-15 |  |  |
| 4 | ＞15 | Diarrhea | Gross bleeding |

**Supplementary Table 2.** The criteria of histology analysis for colonic damage

| Colon damage score | 0 | 1 | 2 | 3 |
| --- | --- | --- | --- | --- |
| Crypt architecture damage | None | Regeneration | Destruction |  |
| Edema in sub-mucosa | None | Mild | Moderate | Severe |
| Inflammatory cells infiltration | None /rare | Lamina propria | Sub-mucosa | Muscle layer |

**Supplementary Table 3.** Primers used for colon tissue qPCR

| Target gene | Primer sequence(5’-3’) |
| --- | --- |
| Gapdh | F: AGGTCGGTGTGAACGGATTTG |
|  | R: GGGGTCGTTGATGGCAACA |
| β-Actin | F: GTGACGTTGACATCCGTAAAGA |
|  | R: GCCGGACTCATCGTACTCC |
| Muc2 | F: TTTCAAGCACCCCTGTAACC |
|  | R: AGGTCCTGGTGTTGAACCTG |
| Occludin | F: CCTCCAATGGCAAAGTGAAT |
|  | R: CTCCCCACCTGTCGTGTAGT |
| ZO-1 | F: CCACCTCTGTCCAGCTCTTC |
|  | R: CACCGGAGTGATGGTTTTCT |
| Claudin-1 | F: TATGTTGGTGCCAGCATTGT |
|  | R: TCATGCCCACCACAGAGATA |
| Claudin-4 | F: GGGGATCATCCTGAGTTGTG |
|  | R: CACTGCATCTGACCTGTGCT |
| TNFα | F: CATCTTCTCAAAATTCGAGTGACAA |
|  | R: TGGGAGTAGACAAGGTACAACCC |
| IL-1β | F: GAGCACCTTCTTTTCCTTCATCTT |
|  | R: TCACACACCAGCAGGTTATCATC |
| IL-10 | F: ATAACTGCACCCACTTCCCA |
|  | R: GGGCATCACTTCTACCAGGT |
| IL-17a | F: TCAGCGTGTCCAAACACTGAG |
|  | R: CGCCAAGGGAGTTAAAGACTT |
| TLR4 | F: GCATGGCTTACACCACCTCT |
|  | R: GTCTCCACAGCCACCAGATT |
| TLR5 | F: CCACCGAAGACTGCGATGAAGAG |
|  | R: CCAGACCTTGTCCTTGAACACCAG |
| NFκB | F: GCTGAGGCACTTCTGAAAGC |
|  | R: AGGTCTGCGTCAAGACTGCT |
| Myd88 | F: CACCTGTGTCTGGTCCATTG |
|  | R: TGAGTGCAAACTTGGTCTGG |
| TRAF6 | F: TCCACACAATGCAAGGAGAA |
|  | R: GTCCATGACCTCTTCGTGGT |
| Cadherin-1 | F: CAGTTCCGAGGTCTACACCTT |
|  | R: TGAATCGGGAGTCTTCCGAAAA |
| Ki-67 | F: ATCATTGACCGCTCCTTTAGGT |
|  | R: GCTCGCCTTGATGGTTCCT |
| PCNA | F: TTGCACGTATATGCCGAGACC |
|  | R: GGTGAACAGGCTCATTCATCTCT |
| β-catenin | F: ATGGAGCCGGACAGAAAAGC |
|  | R: TGGGAGGTGTCAACATCTTCTT |
| STAT3 | F: CACCTTGGATTGAGAGTCAAGAC |
|  | R: AGGAATCGGCTATATTGCTGGT |
| COX2 | F: AGTGGGGTGATGAGCAACTA |
|  | R: GGCAATGCGGTTCTGATACT |

**Supplementary Table 4.** Primers used for fecal microbes DNA qPCR.

| Target gene | Primer sequence(5’-3’) |
| --- | --- |
| Firmicutes | F: GCTGCTAATACCGCATGATATGTC |
|  | R: CAGACGCGAGTCCATCTCAGA |
| Bacteriodetes | F: GAGAGGAAGGTCCCCCAC |
|  | R: CGCTACTTGGCTGGTTCAG |
| Proteobacteria | F: TAGGCTTGACATTGATAGAATC |
|  | R: CTTACGAAGGCAGTCTCCTTA |
| Actinobacteria | F: TGTAGCGGTGGAATGCGC |
|  | R: AATTAAGCCACATGCTCCGCT |
| Parvimonas micra | F: GGTTGTCGTCAGCTCGTGTCGT |
|  | R: TTGCGGTTAGATCGGCGGCTTC |
| Solobacterium moorei | F: ACGTGCAGCGACCTAGCGATAG |
|  | R: ACGGCTCCTTCCTTGCGGTTAG |
| Fusobacterium nucleatum | F: TGAGAGGGTGAACGGCCACAAG |
|  | R: GCGGCTGCTGGCACGTATTTAG |
| Peptostreptococcus stomatis | F: AGCCGCCGAAACTGGAGGACTT |
|  | R: AGCCGCCTTCGCTACTGGTGTT |
| Peptostreptococcus anaerobius | F: AATGCTGCGGTGAATGCGTTCC |
|  | R: ACGACTTCCTCCTTGCGGTTGG |
| Clostridium symbiosum | F: GTGGCGAAGGCGACTTACTGGA |
|  | R: GACGGCACCGAAGAGCTTTGCT |
| Gemella taiwanensis | F: ACGCATTAAGCACTCCGCCTGG |
|  | R: CGACACGAGCTGACGACAACCA |
| Bifidobacterium | F: GATTCTGGCTCAGGATGAACGC |
|  | R: CTGATAGGACGCGACCCCAT |
| Lactobacillus | F: ACGGGAGGCAGCAGTAGGGA |
|  | R: AGCCGTGACTTTCTGGTTGATT |
| 16S rDNA | F: GCAACGAGCGCAACCC |
|  | R: ACGGGCGGTGTGTAC |
